# Impaired Cerebrovascular Function in Coronary Artery Disease Patients and Recovery Following Cardiac Rehabilitation

**DOI:** 10.3389/fnagi.2015.00224

**Published:** 2016-01-05

**Authors:** Udunna C. Anazodo, J. K. Shoemaker, Neville Suskin, Tracy Ssali, Danny J. J. Wang, Keith S. St. Lawrence

**Affiliations:** ^1^Lawson Health Research Institute, LondonON, Canada; ^2^Department of Medical Biophysics, Western University, LondonON, Canada; ^3^Laboratory for Brain and Heart Health, School of Kinesiology, Western University, LondonON, Canada; ^4^London Health Sciences Cardiology Rehabilitation Program, LondonON, Canada; ^5^Department of Neurology, David Geffen School of Medicine, University of California, Los Angeles, Los AngelesCA, USA

**Keywords:** arterial spin labeling (ASL), cerebral vascular reactivity (CVR), coronary artery disease, cerebral blood flow (CBF), aerobic exercise, cardiac rehabilitation

## Abstract

Coronary artery disease (CAD) poses a risk to the cerebrovascular function of older adults and has been linked to impaired cognitive abilities. Using magnetic resonance perfusion imaging, we investigated changes in resting cerebral blood flow (CBF) and cerebrovascular reactivity (CVR) to hypercapnia in 34 CAD patients and 21 age-matched controls. Gray matter volume (GMV) images were acquired and used as a confounding variable to separate changes in structure from function. Compared to healthy controls, CAD patients demonstrated reduced CBF in the superior frontal, anterior cingulate (AC), insular, pre- and post-central gyri, middle temporal, and superior temporal regions. Subsequent analysis of these regions demonstrated decreased CVR in the AC, insula, post-central and superior frontal regions. Except in the superior frontal and precentral regions, regional reductions in CBF and CVR were identified in brain areas where no detectable reductions in GMV were observed, demonstrating that these vascular changes were independent of brain atrophy. Because aerobic fitness training can improve brain function, potential changes in regional CBF were investigated in the CAD patients after completion of a 6-months exercise-based cardiac rehabilitation program. Increased CBF was observed in the bilateral AC, as well as recovery of CBF in the dorsal aspect of the right AC, where the magnitude of increased CBF was roughly equal to the reduction in CBF at baseline compared to controls. These exercise-related improvements in CBF in the AC is intriguing given the role of this area in cognitive processing and regulation of cardiovascular autonomic control.

## Introduction

Coronary artery disease (CAD) is the most prevalent form of cardiovascular disease, encompassing acute coronary syndromes that are characterized by atherosclerotic lesions in coronary arteries which impair myocardial blood flow. It is estimated that at the age of 40, one in two men and one in three women will develop CAD (Lloyd-Jones et al., 1999), representing the highest lifetime risks for any disease (Lloyd-Jones et al., 2006). By contrast, at the age of 50, the lifetime risk for the most common type of cancers, breast cancer in women and prostate cancer in men, is 12 and 15%, respectively (Howlader et al., 2013). Although higher CAD survival rates are now achieved, well into the third decade of the disease (Lloyd-Jones et al., 2006), age-related chronic diseases such as CAD can pose a threat to an individual’s ability to maintain a high mental and physical functional capacity. While, considerable efforts are being made to improve the physical functional capacities of CAD patients, including interventions, such as cardiac rehabilitation (CR), aimed at minimizing recurrence risks and improving overall cardiovascular functions (Clark et al., 2005), recent evidence points to the possible comorbidity of CAD and neurological impairments which threaten the mental capacity of CAD patients (Friedman et al., 2014). A number of studies have demonstrated that risk factors for CAD are also associated with accelerated brain decay and alterations to the natural age-related decline in cerebrovascular functions (de Toledo Ferraz Alves et al., 2010; Friedman et al., 2014). Recently, we observed significant reductions in regional brain volume in CAD patients (Anazodo et al., 2013) that are consistent with observations of cerebral atrophy, hypoperfusion, and white matter (WM) disease in brains of older adults with increased risk for vascular disease (Friedman et al., 2014), and match regions associated with cognitive decline in cardiovascular patients (Okonkwo et al., 2010). Although these observations suggest a possible link between CAD and neurological dysfunction, the interaction of age, vascular disease and cerebrovascular dysfunction is complex and not well-understood (de la Torre, 2000; de Toledo Ferraz Alves et al., 2010). Furthermore, no study to date has assessed the potential impact of CAD on regional cerebrovascular function and hemodynamics in older adults. Identifying such changes would improve the understanding of cardio-cortical interactions and highlight the added benefits to cerebrovascular health provided by CR.

Interestingly, when older adults with CAD performed aerobic exercise training, typically included in CR programs, enhanced brain structure and cognitive performance has been observed. For instance, in the same cohort of CAD patients, where we first reported decreased brain volume, an increase in regional brain volume was seen after completion of a 6-months exercise-based CR program (Anazodo et al., 2013). Improved performance on tasks for executive function, psychomotor speed and episodic memory have been demonstrated by a few studies in cardiovascular disease patients that performed structured exercise training during a 12–18 weeks CR program (Hayes et al., 2014). It still remains to be seen if exercise-based CR programs can enhance cerebrovascular function in CAD patients as well. Based on these observations and building on our previous work, the objectives of this study were to investigate changes in cerebrovascular function in CAD patients and assess if potential changes would be related to regional brain atrophy. Measurements of cerebral blood flow (CBF) and cerebrovascular reactivity to CO_2_ (CVR) were acquired using arterial spin labeling (ASL), a non-invasive magnetic resonance imaging (MRI)-based perfusion technique capable of measuring both resting CBF and flow changes in response to alterations in arterial CO_2_ tension (i.e., CVR; Tancredi et al., 2015). We tested the hypothesis that CBF and CVR would be reduced in cognitive areas of the brains of CAD patients, since increased vascular resistance, common in CAD can diminish CVR (Novack et al., 1953). As a corollary, we hypothesized that aerobic exercise training performed as part of CR would attenuate disease effects. To ensure regional changes in CBF and CVR were independent of underlying changes in brain structure, gray matter volume (GMV) measurements described in the earlier study (Anazodo et al., 2013), were used as confounding variables.

## Materials and Methods

### Participants

This study was approved by the Western University Health Sciences Research Ethics Board and written informed consent was obtained from all participants. The present sample were subjects from a previous study (Anazodo et al., 2013) and consists of 34 CAD patients (58 ± 7 years, 10 females) and 21 healthy controls (59 ± 8 years, 10 females) whom had MRI scans that included ASL acquisitions. Of the 34 patients, 17 (59 ± 6 years, five females) completed a 6-months CR program and participated in post-CR testing. A 50% CR participation rate is common in hospital-based CR programs (Mampuya, 2012). The CAD patients were recruited from the London Health Sciences Center for Cardiac Rehabilitation and Secondary Prevention program, and controls matched in age, were recruited from the local community. The CAD group comprised of individuals with clinical diagnosis for CAD including acute coronary syndromes such as ST elevation and non-ST elevation myocardial infarct, and angina pectoris confirmed by coronary angiography or standard non-invasive diagnostic tests such as echocardiography, exercise stress testing and myocardium perfusion testing. Exclusion criteria included patients with congenital coronary abnormality, cardiomyopathy, severe congestive heart failure, second or third-degree atrioventricular block, more than two myocaridal infarcts, sick sinus syndrome, complicated arrythmias, uncontrolled hypertension (sustained blood pressure higher than 140/90 mmHg despite use of three antihypertensive agents at maximum dosage; Calhoun et al., 2008) or a history of diabetes for more than 5 years. Patients who had percutaneous coronary intervention or coronary artery bypass surgery within 3 months of the study were excluded to avoid potential transient post-operative neurocognitive complications (Rasmussen, 2006). All participants were free of neurological diseases, pharmaceutical or nutraceutical psychostimulants, nootropics and were non-smokers.

### Experimental Design

Experimental data were collected over three testing sessions: (1) a laboratory session for assessment of clinical markers of vascular health, (2) graded exercise testing, and (3) brain MRI. Subjects refrained from caffeine, alcohol and physical activity at least 12 h before participating in each session. To capture global cognitive function, the Montreal Cognitive Assessment (Nasreddine et al., 2005; MoCA), was administered during the laboratory testing. MoCA scores were corrected for level of education – defined by the total number of years of formal education (Nasreddine et al., 2005). The entire experimental protocol was repeated in the 17 CAD patients who performed 6 months of CR, which consisted of moderate-intensity aerobic and strength fitness training. The aerobic training protocol was performed as previously described (Anazodo et al., 2013), as part of the London Health Sciences Center Cardiac Rehabilitation and Secondary Prevention Program.

Cerebrovascular reactivity measurements were achieved by manipulations of arterial concentrations of CO_2_. Subjects breathed room air for 5 min, followed by 5 min inhalation of 6% CO_2_ mixed with 21% O_2_ and balanced nitrogen, while end-tidal partial pressure of CO_2_ (P_ET_CO_2_) was recorded continuously (Invivo 3150m, Invivo Corp., Orlando, FL, USA). Air and mixed CO_2_ were delivered via a non-rebreathing facemask (Hans Rudolph, Inc., Kansas City, MO, USA) attached to a large non-diffusible gas reservoir bag. The breathing rate was paced at 15 breaths per minute guided by a metronome to minimize hyperventilation and maintain steady-state sampling of P_ET_CO_2_. The hypercapnia challenge was performed first in the laboratory in supine position to acclimatize participants prior to repeating the challenge during brain imaging. Participants unable to perform hypercapnia testing or who had a change in P_ET_CO_2_ (ΔP_ET_CO_2_) of 5 mmHg or less in the laboratory examinations did not perform the challenge during the MRI session. Instead only resting CBF images were acquired.

### Clinical Measurements

To assess levels of plasma lipids, cholesterol, blood glucose, glycated hemoglobin and high-sensitivity C-reactive protein, blood samples were collected under fasting conditions and analyzed using standard assays and glucometer. Three-lead electrocardiogram and finger blood pressure monitoring (Finometer; Finapres Medical Systems, Amsterdam, the Netherlands; calibrated to sphymomanometric values) were performed concurrently over a 20 min period. Mean values for heart rate, systolic blood pressure, diastolic blood pressure, and mean arterial pressure were reported from measures averaged over 5 min. Vascular hemodynamic measurements were performed on the right common carotid artery using Doppler ultrasound imaging (Vivid 7, GE Healthcare). Carotid vessel diameter, wall thickness and intima media thickness were measured from 2-dimensional (2D) B-mode images acquired in the long-axis plane by a single observer blinded to subject identity or group assignment. Arterial compliance and distensibility were calculated from measures of vessel diameter and pressure at end systole and end diastole (Nielson et al., 2014). A 2D transthoracic Doppler echocardiogram (Vingmed System FiVe, GE Healthcare, Chalfont St Giles, UK) was performed to assess left ventricular ejection fraction - a clinical index of cardiac function. Graded exercise testing was performed to volitional exhaustion to measure cardiorespiratory fitness level or capacity (maximal oxygen consumption, VO_2_ max), in accordance with the American College of Sports Medicine guidelines for exercise testing.

### MRI Data Acquisition

All MRI brain imaging was performed on a Siemens 3T Verio system (Siemens Medical Systems, Erlangen, Germany) using a 32-channel head coil. The head was immobilized with foam padding to minimize motion artifacts. Sagittal T1-weighted images were acquired using a three-dimensional magnetization-prepared rapid gradient-echo (MPRAGE) sequence [repetition time (TR), echo time (TE) and inversion time (TI) = 2000, 2.98, and 900 ms, flip angle = 9°, field of view (FOV) = 256 mm × 256 mm, 176 slices, isotropic voxel size = 1.0 mm^3^, acceleration factor = 3]. ASL images were acquired during hypercapnia testing using a transverse 2D gradient-echo echo planar imaging (EPI) sequence (TR/TE = 3500/12 ms, FOV = 240 mm × 240 mm, 12 contiguous slices, 6 mm thickness, voxel size = 3.8 mm × 3.8 mm × 6 mm, bandwidth = 2298 Hz/Px, acceleration factor of 2) and a pseudo-continuous label method (Wang et al., 2003) applied 9 cm below the center of the imaging volume for a duration of 1.5 s. One-hundred and ten label and control pairs were acquired after a 1.0 s post-label delay. A non-selective inversion pulse was applied for background suppression during the post-label delay. To improve signal-to-noise (SNR) and sensitivity of ASL for CVR measurements, ASL imaging and hypercapnia testing were repeated after a 3 min recovery period. Additional imaging for CBF quantification included; (a) proton density calibration scan (M_0_) acquired with the ASL sequence using a TR of 7 s and no label or background suppression pulses, and (b) axial single-shot inversion-recovery prepared balanced steady-state free precession imaging (IR-TrueFISP) acquired with 10 variable TI values (175–8000 ms) on a single slice at the mid-sagittial section of the brain (TE = 1.21 ms, 1.7 mm × 1.7 mm × 4 mm, acceleration factor of 2). These images were used to measure the longitudinal relaxation time of blood (T1_b_) in the sagittal sinus (Xu et al., 2010).

### Perfusion-weighted Analysis

Preprocessing and analysis of perfusion data were performed with SPM8^1^ and scripts written in MATLAB (2012a, The MathWorks, Natick, MA, USA). All ASL and M_0_ scans were aligned to the first time point of the first scan to correct for head motion within sessions (two trials) and between sessions (pre and post-CR). Intra-subject alignment was performed on MPRAGE images using the VBM8 longitudinal toolbox^2^ in SPM8 to minimize intra-subject registration errors. Using a rigid-body transformation, respective MPRAGE image volumes were aligned to corresponding M_0_ and ASL scans (i.e., baseline MPRAGE to baseline ASL and post-CR MPRAGE to post-CR ASL), and segmented into gray matter (GM), WM, and cerebrospinal fluid (CSF) probability images using the new segment tool in SPM8. The GM segments were transformed into binary masks thresholded for voxels with 80% or more GM content and applied to the ASL time series. This was done to reduce the ASL signal contributions from WM and CSF. Forty label and control images were extracted from each 5 min block at steady-state. Surround subtraction and time-averaging over the two trials were used to generate mean perfusion-weighted (ΔM) images. CBF was calculated using a standard single-compartment flow model (Wang et al., 2003).

$$CBF(ml/100g/\min)=\frac{6000.\lambda .\Delta M.e^{\left(\frac{\omega }{{T1}_{b}}\right)}}{2\alpha .M_{0}.{T1}_{b}.\left(1-e^{\left(-\frac{\tau +\omega }{{T1}_{b}}\right)}\right)}$$

where aaa = blood/tissue water partition coefficient, 0.9 g/mL; α = labeling efficiency assumed to be 85% (Xu et al., 2010) multiplied by 94% for background suppression; ω = post-labeling delay of 1.0 s incremental per slice; τ = label duration of 1.5 s and T1_b_ = individual blood T1 value. The final individual normocapnia and hypercapnia CBF images were smoothed using a Gaussian filter with a FWHM of 6 mm and transformed to standard stereotactic space (MNI) using transformation parameters from segmentation of MPRAGE images. CVR was calculated pixel-by-pixel as the increase in CBF per mm Hg increase in P_ET_CO_2._ All further reference to CBF refers to CBF measured at room air, unless otherwise stated.

To assess the sensitivity of the ASL sequence used in this study, the temporal signal-to-noise ratio (tSNR) of the perfusion-weighted time series was calculated for each subject. Temporal SNR was defined as the mean GM pixel signal relative to the mean GM pixel standard deviation. The reproducibility of repeated ASL measurements was determined from a subset of patients and controls (*N* = 19) for baseline CBF and CVR using within-sessions coefficient of variation and intraclass correlation coefficient (ICC). ICC was calculated using SPSS and two-way random model with measures of consistency, where a value close to 1 represents a high reliability. For completeness, the test–retest reliability at baseline was also compared voxel-by-voxel using repeated measures analysis of variance. This was done to ensure that averaging the perfusion-weighted signal from the two trials did not bias group comparisons.

### Assessment of Disease Effects

**FIGURE 1:**
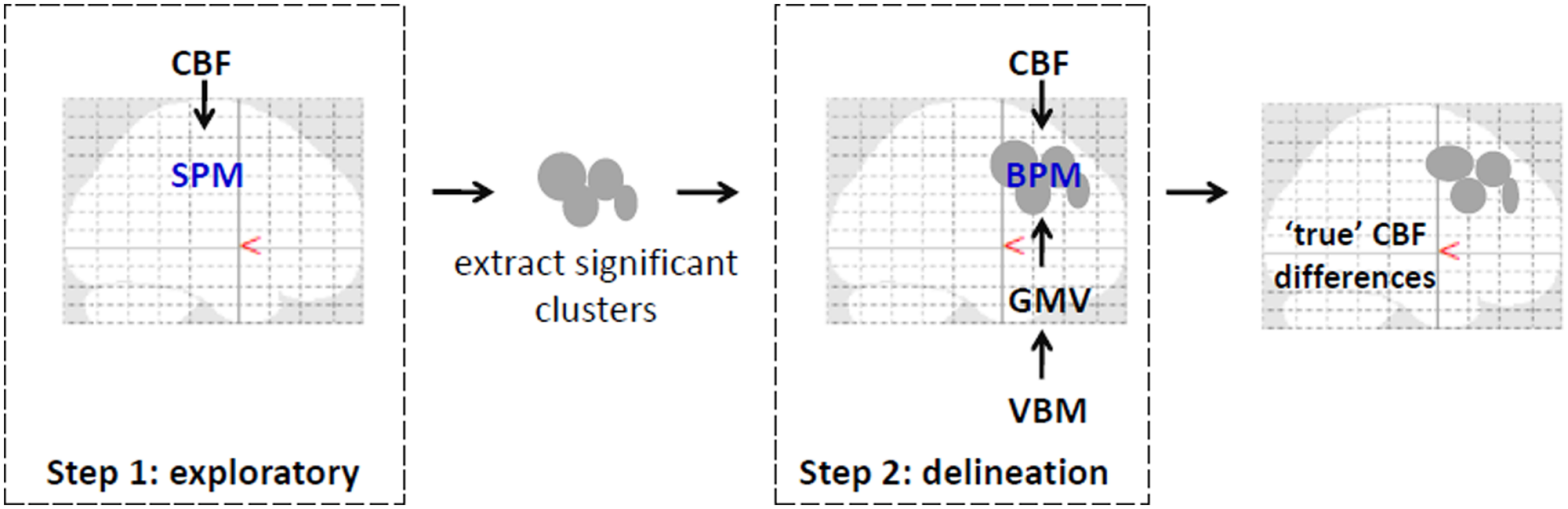
**An illustration of the pipeline for multimodal voxel-wise mass-univariate analysis of variance performed to determine the singular effect of CAD on regional CBF.** Step 1 involved SPM voxel-wise between-group comparisons of CBF images across the brain. Significant clusters from this step were converted to binary masks and used in step 2 to improve voxel-wise multimodal analysis and limit type II errors. In step 2, between-group differences in CBF were examined voxel-wise using BPM with GMV images from VBM analysis serving as covariates. Corrections for multiple comparisons using FDR (*p* < 0.05) was performed at each step. Clusters that remained signified regions where CBF changes drive the observed differences as in **Figure 3** and as listed in **Table 2**. Illustration was overlaid on glass brains from SPM8 (http://www.fil.ion.ucl.ac.uk/spm/).

To delineate perfusion changes from underlying changes in brain volume (Anazodo et al., 2013) on a voxel-by-voxel basis, a multimodal mass-univariate analysis was performed as a two-step process as outlined in **Figure 1**. First, an exploratory analysis was performed on the CBF images across all voxels with greater than or equal to 80% GM, to identify regions with significantly different GM CBF between CAD patients and age-matched controls. This was achieved using two-tailed Student’s *t*-test performed within SPM8. Next, these clusters were passed to the biological parametric mapping (BPM; Casanova et al., 2007) tool, where a voxel-by-voxel analysis of covariance (ANCOVA) was performed on the CBF images with differences in regional GMV removed. This resulted in differences in regional GM perfusion that were independent of GMV changes.

For the BPM analysis, individual GMV images were generated from MPRAGE volumes using voxel-based morphometry (VBM^3^) in SPM8 as described in a prior study (Anazodo et al., 2013). The GM segments were spatially normalized to a template averaged from all subjects, corrected for differences in brain size and smoothed with an 8-mm Gaussian filter. To describe changes in GMV within the clusters of interest, a voxel-by-voxel between-group comparison was performed on the GMV images, restricted to the clusters of interest. An absolute threshold mask set at 0.1 was used to remove non-GM voxels. For all statistical analysis, type I errors were minimized using false discovery rate (FDR) at *p* < 0.05 and cluster size greater than 10 voxels.

Since, lower CVR was expected in the CAD (Novack et al., 1953) group compared to controls, a one-tailed *t*-test was performed on regional mean CVR values extracted from each of the clusters from results of ANCOVA BPM analysis. Voxel-by-voxel comparisons were not performed on CVR images because of power constraints. The CVR data of one patient were excluded due to accelerated breathing (>25 breathes per minute) during hypercapnia, which can confound the CVR results.

### Assessment of Cardiac Rehabilitation Effects

A paired sample *t*-test was performed voxel-by-voxel between baseline and post-CR CBF images of CAD patients using SPM. Areas of significant differences in CBF were identified using a small volume correction approach within *a priori* regions of interest (ROI). Two anatomical ROI, each in the right and left anterior cingulate (AC) cortex were derived using the automated anatomical labeling atlas (Tzourio-Mazoyer et al., 2002) in WFU PickAtlas (Maldjian et al., 2003) toolbox, because in older adults the AC is known to display robust changes in brain activity in response to exercise training (Burdette et al., 2010; Chapman et al., 2013; Wong et al., 2015). The GMV images were not included as covariates since no change in GMV were observed in the AC of the patients’ post-CR (Anazodo et al., 2013). Using the MarsBaR ROI toolbox^4^ *a priori* ROI masks were created from regions of increased GMV post-CR reported in an earlier study (left and right medial frontal gyri; Anazodo et al., 2013). Functional ROI masks derived from results of baseline ANCOVA BPM analysis were also included in the small volume correction analysis to evaluate areas of CBF recovery with CR. This was further demonstrated using percent relative change computed from individual regional means extracted from baseline results. Baseline percent changes were relative to each regional mean CBF across all control subjects, while post-CR percent changes were relative to pre-CR regional CBF values. For completeness, percent changes were also computed for GMV using the functional ROI masks. Analyses was not performed on post-CR CVR data due to a lack of statistical power.

### Statistical Analysis

Statistical analyses were conducted with SPSS 20.0 statistical software (IBM Corp., Armonk, NY, USA). Baseline clincal assesments of CAD patients were compared to data from the control group using two-tailed Student’s *t*-test since age and gender were matched. To test for effects of aerobic fitness, a paired *t*-test was performed on clinical data acquired on a subgroup of CAD patients before and 6 months after CR.

## Results

### Study Demographics

**FIGURE 2:**
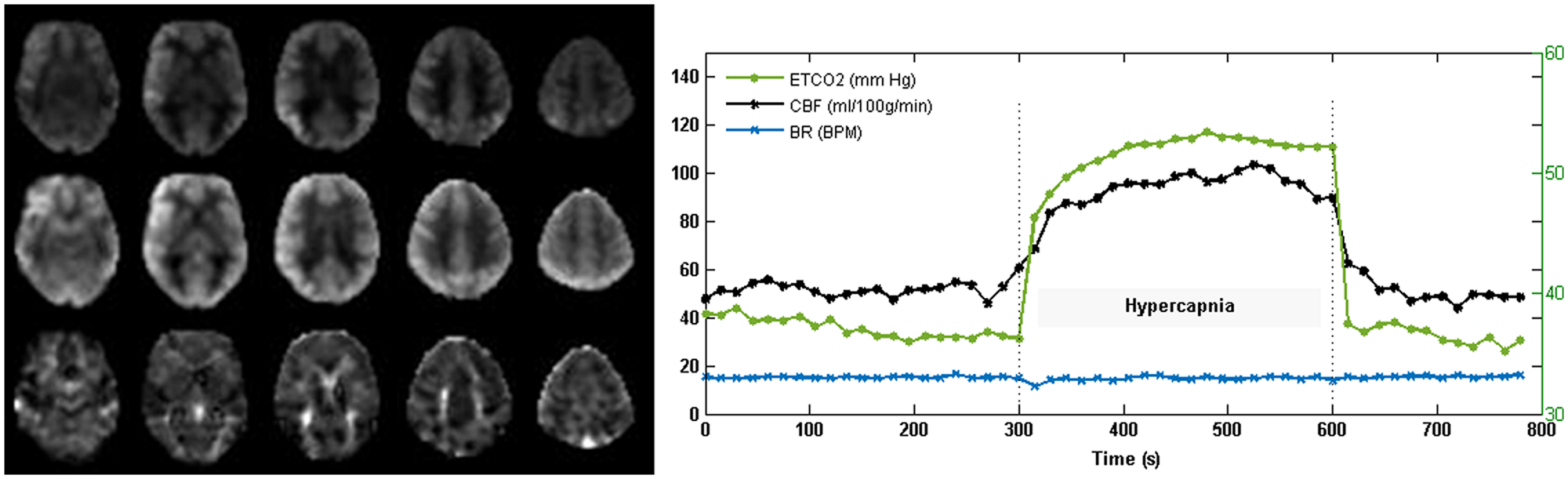
**Cerebral blood flow (CBF) and CVR images acquired with pCASL from one subject.** The representative CBF (normocapnia; top and hypercapnia; middle) and CVR (bottom) maps demonstrate good gray to white matter contrast in parenchymal CBF and no evidence of intravascular artifacts or signal dropouts. Time courses of P_ET_CO_2_ and total gray matter CBF show the expected CBF response to capnia. Breathing rate (BR) in breaths per minute (BPM) was maintained at 15 breathes per minute (BPM).

Perfusion data from one control subject and one CAD patient at baseline were excluded because of motion artifacts. Thirteen control subjects and 22 CAD patients participated in CVR measurements. Axial images of CBF during normocapnia and hypercapnia, and CVR from a representative subject along with time series of P_ET_CO_2_, breathing rate and mean whole brain pCASL signal are displayed in **Figure 2**. The CAD patients were on a combination of drug therapy to lower lipid levels (statins = 83.3%), maintain blood pressure (beta-blockers = 72.2%; ACE-inhibitors/angiotensin II receptor blockers = 50%) and prevent reinfraction (anti-platelets/aspirin = 77.8%). Forty-one percent had precutaneous coronary intervention and 8.8% received coronary artery bypass grafting prior to participation in the study.

### Baseline Clinical Measures

Results of clinical assessments compared between groups at baseline including global means of GM CBF and CVR are presented in **Table 1**. There were no significant differences between patients and controls in terms of blood pressure, left ventricular function, resting heart rate and blood glucose. CAD patients had lower total cholesterol [*F* = 15.34 (1,53), *p* < 0.0001] likely reflecting the therapeutic effect of the combined drug therapy received by patients. However, CAD patients had lower MoCA scores [*F* = 4.63 (1,51), *p* < 0.01] after adjustment for level of education, lower VO_2_ max [*F* = 15.02 (1,37), *p* < 0.0001], elevated BMI [*F* = 18.46 (1,53), *p* < 0.0001], and higher carotid artery intima media thickness [*F* = 8.05 (1,43), *p* < 0.001]. There was also a trend of reduced compliance in the common carotid (*p* = 0.24) and elevated hs-CRP (*p* = 0.10), a marker of inflammation and measure of myocardial infarction risk.

**Table 1:** Study demographics.

Variables	Controls	Patients
Body mass index (kg/m^2^)	24.54 3.11	29.84 4.73ˆ*
Glucose (mmol/L)	4.72 0.89	5.21 1.38
Total cholesterol (mmol/L)	4.15 0.96	3.09 0.79ˆ*
High-sensitivity C-reactive protein (mg/L)	0.97 0.91	2.10 3.10
Systolic blood pressure (mm Hg)	120.28 15.97	126.70 21.20
Diastolic blood pressure (mm Hg)	68.18 7.8	71.5 12.41
Heart rate (beats per min)	58.34 9.54	59.11 6.85
Left ventricular ejection fraction (%)	67.41 9.82	64.35 7.24
Intima media thickness, carotid (mm)	0.53 0.12	0.64 0.12ˆ*
Compliance, carotid (mm/mm Hg)	0.0088 0.003	0.0077 0.003
MOCA	28.32 1.67	26.82 2.1ˆ*
VO_2_ max (mL/kg/min)	37.27 9.94	26.9 7.24ˆ*
ΔP_ET_CO_2_ (mm Hg)	11.35 3.06	13.09 3.67
Global mean CBF (mL/100 g/min)	50.83 11.68	46.49 13.88
Global mean CVR (mL/100 g/min/mm Hg)	2.57 0.62	2.26 0.63


*Subject characteristics. Means and standard deviations of clinical assessments compared between CAD patients (N = 33) and controls (N = 20). Statistical differences between groups at p<0.05 are indicated by ^∗^.*

### Baseline Brain Imaging

**FIGURE 3:**
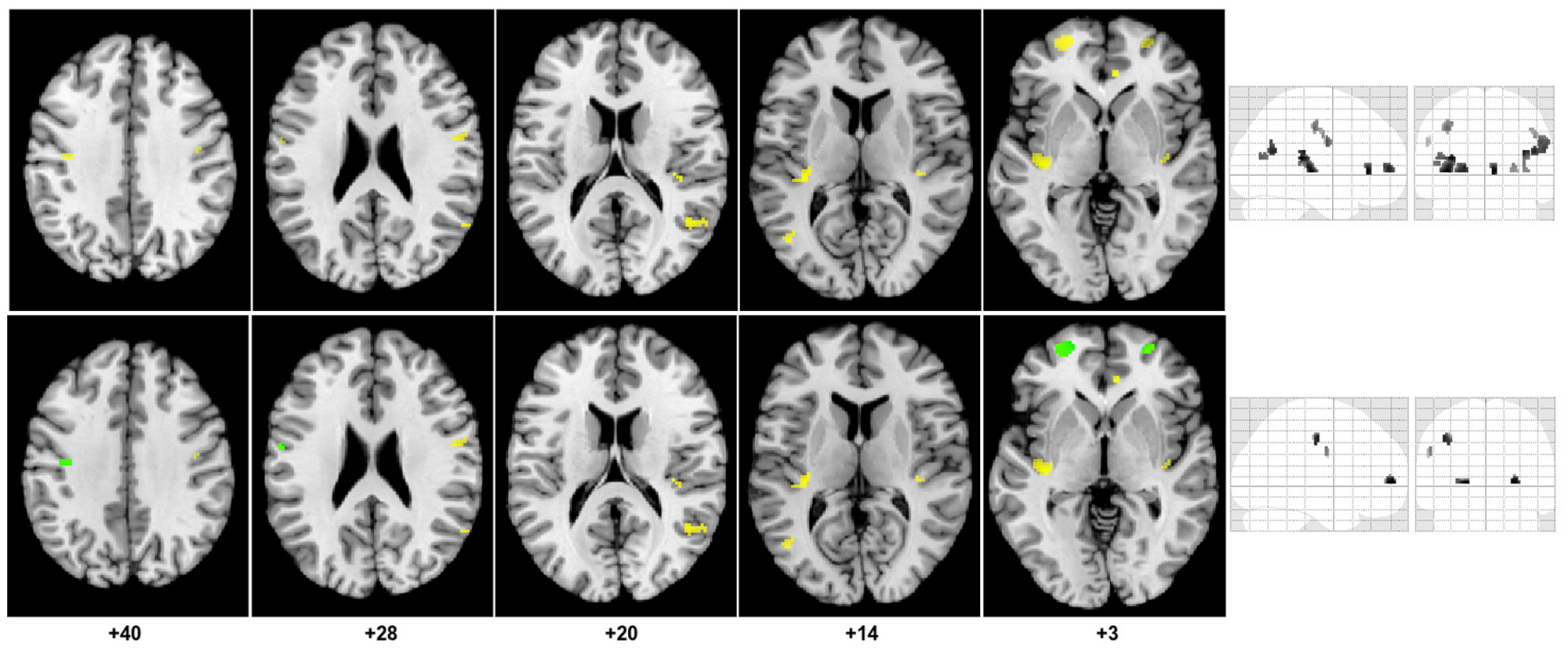
**Regions of decreased gray matter CBF in CAD patients compared to controls.** The top portion of the figure shows clusters (yellow) of decreased gray matter CBF overlaid on the axial slices of the Colin27 brain template (MNI) and on a coronal and sagittal slice of the SPM glass brain. Concomitant decrease in GMV and gray matter CBF are shown in the bottom portion of the figure.

Mean T1_b_ across all subjects was 1563 ± 70 ms. There was no difference between T1_b_ for patients and controls. Mean GM tSNR for the ASL-EPI sequence was 4.0 ± 1.4. Test–retest reliability of the CBF and CVR measurements performed voxel-wise, showed no differences between trials (*p* < 0.001, uncorrected). The ΔM signals were averaged over the two trials because of the reproducibility of GM CBF and CVR. The CV = 6.4% for CBF and 14.9% for CVR with corresponding ICC (2, 1) values of 0.93 and 0.82 for CBF and CVR respectively. No differences were found in global mean CBF or CVR between CAD patients and controls. Results from the ANOCVA BPM analysis are listed in **Table 2** and are represented graphically on axial slices through the brain in **Figure 3**. The results were as follows:

**Table 2:** Results of multimodal voxel-by-voxel mass-univariate analysis.

Brain region (Brodmann area)		Talairach coordinate (X, Y, Z)			# of voxels	SPM t	SPM p	BPM t	BPM p
**Regional gray matter CBF, controls > patients**								
(1) L Superior temporal (41)		-34	-30	14	119	5.64	0.01	5.20	<0.001
L Insula (13)		-38	-23	3		5.03	0.04	4.97	<0.001
(2) L Superior frontal (10)		-22	50	-1	63	5.11	0.01	4.57	<0.001
(3) R Anterior cingulate (24)		6	31	0	18	4.91	0.01	4.98	<0.001
(4) L Post-central gyrus (3)		-38	-20	42	36	4.79	0.01	3.55	<0.001
(5) R Post-central gyrus (6)		53	-6	28	35	4.71	0.01	3.95	<0.001
(6) R Middle temporal (39)		46	-62	20	57	4.47	0.02	4.50	<0.001
R Superior temporal (39)		55	-57	23		4.23	0.03	4.15	<0.001
(7) R Insula (13)		36	-28	18	33	4.25	0.03	3.36	<0.001
(8) R Superior frontal (10)		26	49	-1	21	4.21	0.03	3.23	0.002
(9) ^†^L Precentral gyrus (4)		-55	-8	28	14	4.18	0.03	2.49	0.008
(10) R Precentral gyrus (6)		46	-10	28	10	4.11	0.03	3.01	0.003
(11) L Middle temporal (39)		-46	-69	16	18	3.61	0.04	3.69	<0.001
**VBM: Regional GMV, controls > patients**								
(1) R Superior frontal (10)		25	50	0	21	3.97	0.007		
(2) L Superior frontal (10)		-22	51	1	42	3.95	0.007		
(3) L Post-central gyrus (4)		-41	-16	39	31	3.69	0.007		
(4) L Precentral gyrus (4)		-54	-9	32	14	3.04	0.012		


*Results of voxel-by-voxel comparisons in regional cerebral blood flow and in regional gray matter volume between CAD patients (N = 33) and control (N = 20). Effect magnitude is represented by t-static from SPM analysis and from BPM analysis where changes in gray matter volume were accounted for. Results were corrected for multiple comparisons (FDR, p<0.05). Coordinates of local maxima of clusters of significant difference are listed and given in anatomical Talairach space. R = right; L = left; ^†^for the left precentral gyrus, the total number of voxels from BPM analysis was reduced to 12.*

(1)  Eleven clusters were identified as having significantly lower CBF in the CAD patients from the SPM exploratory step. The corresponding anatomical labels are listed in **Table 2**.
(2)  All 11 clusters remained significant after controlling for differences in GMV between the two groups. However, the magnitude of the effect of disease on CBF observed after atrophy correction was slightly muted in nearly all regions, except in the right AC cortex and bilateral middle temporal gyri (**Table 2**).
(3)  The cluster size of nearly all regions was also preserved after BPM ANCOVA analysis except in the left precentral gyrus where the cluster size was reduced by 2 voxels.
(4)  Within the 11 regions, a significant decrease in GMV was observed in the CAD group in the right and left superior frontal, left post-central gyrus and left precentral gyrus. These four regions also had the greatest reductions in CBF changes following BPM analysis, as indicated by reduced *t*-value (**Table 2**).

In general, the brains of CAD patients had significant reductions in regional GM CBF independent of GMV in the bilateral prefrontal, insula, middle temporal, superior temporal, post-central gyri, and in the right AC cortex. Concomitant decrease in GMV and atrophy-independent CBF were observed in the right and left superior frontal, and in the left pre- and post-central gyri.

**FIGURE 4:**
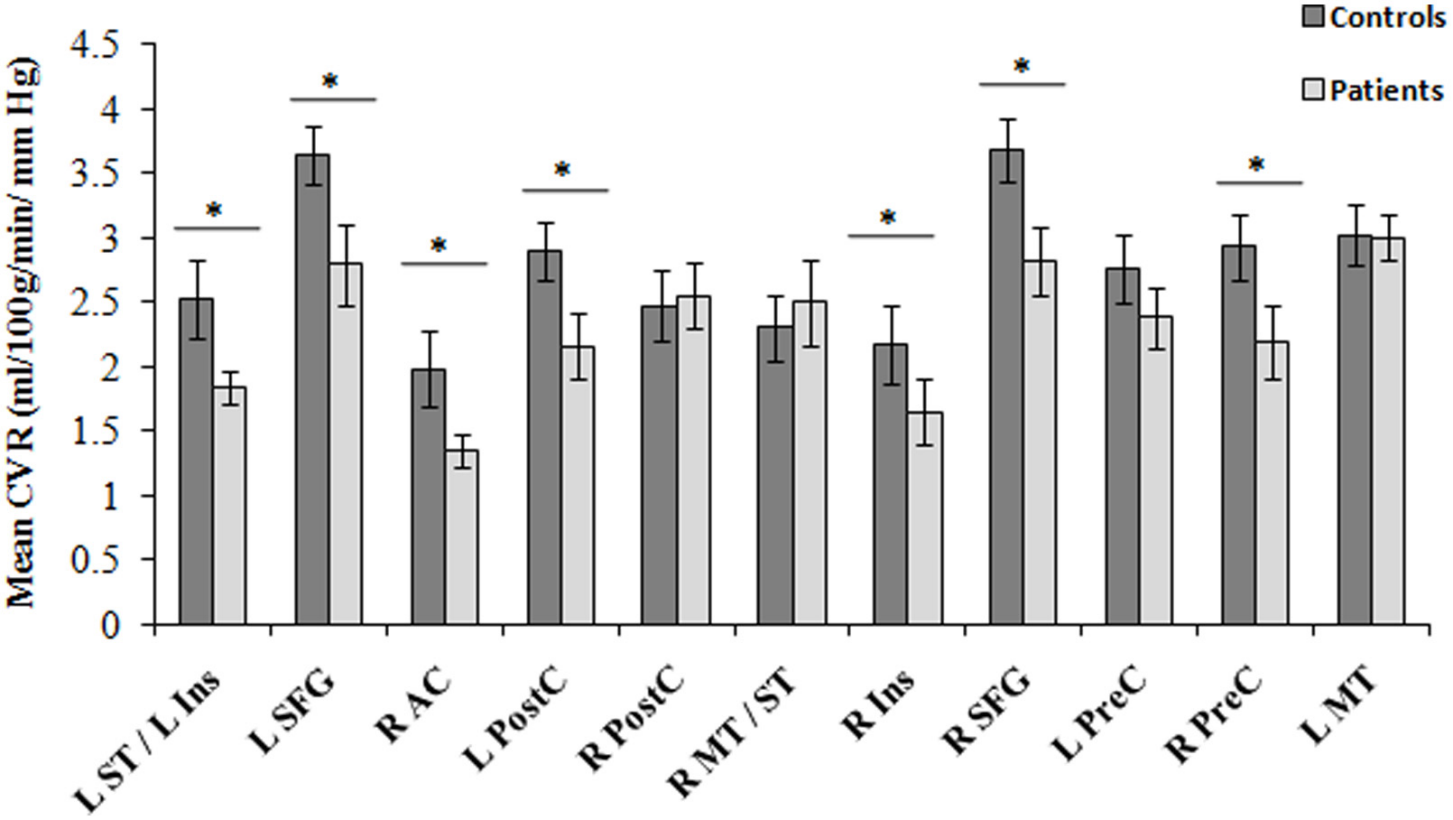
**Regions of differences in regional CVR compared between CAD patients (*N* = 22) and controls at baseline (*N* = 13).** Statistical differences between groups at *p* < 0.05 are signified by ^∗^ and error bars indicate standard errors of the mean. R, right; L, left; SFG, superior frontal gyrus; ST, superior temporal gyrus; Ins, insula; PostC, post-central gyrus; MT, middle temporal gyrus; PreC, precentral gyrus; AC, anterior cingulate.

Between-group comparisons in regional CVR are shown in **Figure 4**. Group means from each cluster and the standard error of the means are displayed. Significant decreases in CVR were observed in the CAD patients in the right AC, bilateral superior frontal gyrus, left superior temporal/insula, left post-central gyrus, right insular and right precentral gyrus (all, *p* < 0.05).

### Cardiac Rehabilitation Effects

**FIGURE 5:**
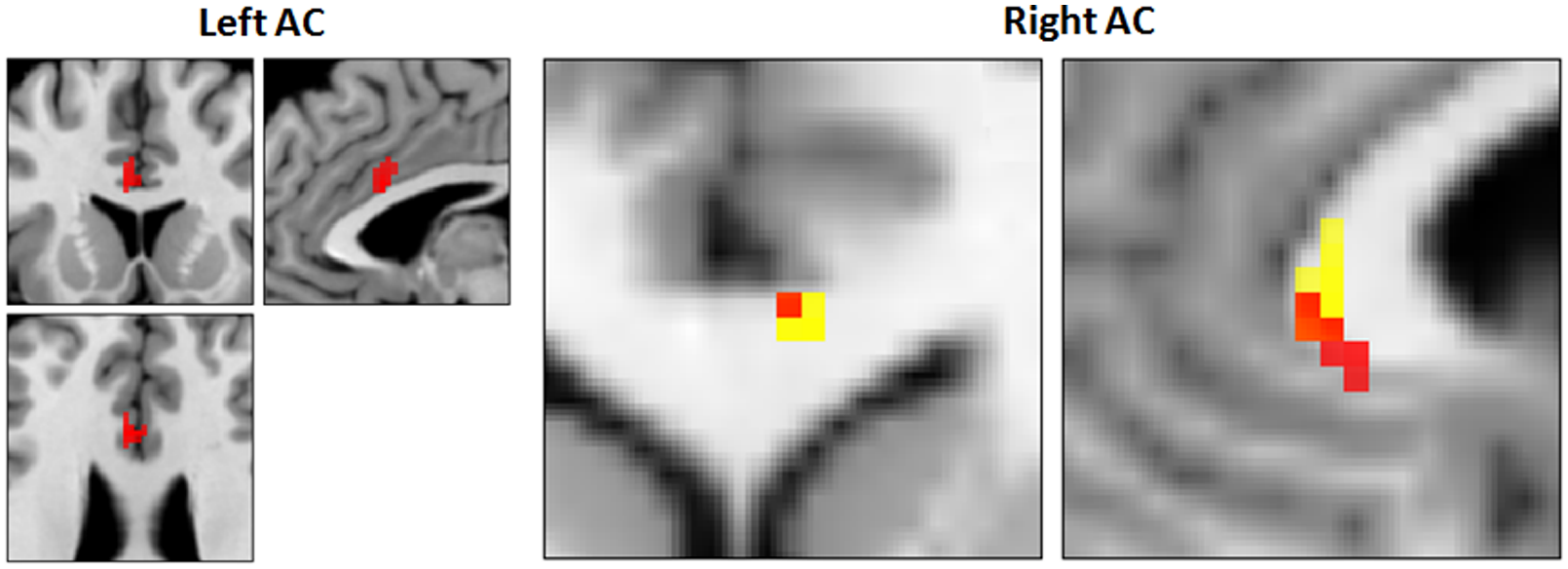
**Increased CBF and recovery in CAD patients post-CR (*N* = 17).** Clusters of higher CBF in right and left anterior cingulate (AC) are shown in red. Conjunction analysis identified recovery of CBF post-CR in a focal area in the right AC (orange, 5; 29; 3, Talairach coordinates) that overlapped with a region of decreased CBF at baseline (yellow).

**FIGURE 6:**
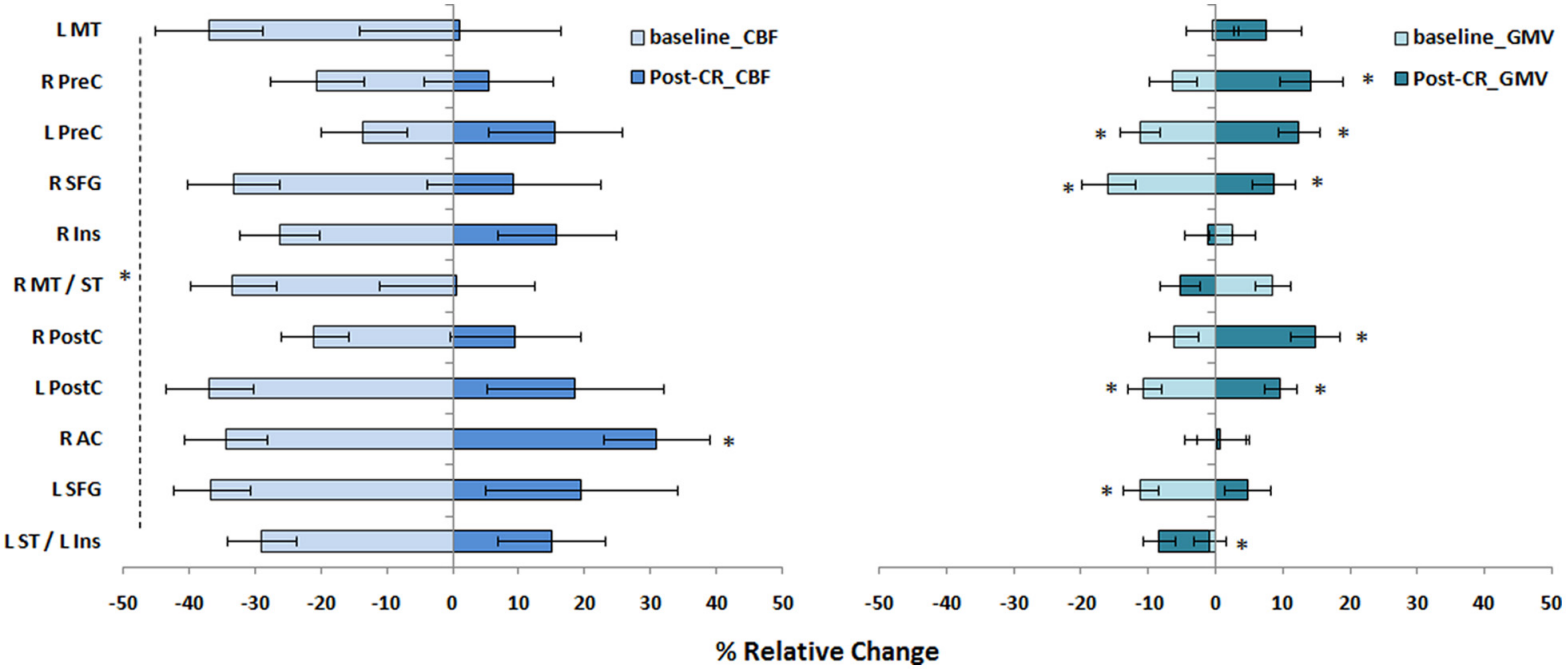
**Magnitude of regional CBF changes in CAD patients.** The magnitude of changes in CBF and GMV in CAD patients at baseline and recovery after CR are shown here as a relative change. Regions were derived from results of baseline comparisons between patients and controls (**Table 2**). Statistical differences at *p* < 0.05 are signified by ^∗^ and error bars indicate standard errors of the mean. See **Figure 4** footnote for full name of regions.

There were no differences within the CAD patient group in pre- and post-CR clinical tests. Mean VO_2_ max at baseline and post-CR were 28.04 ± 10.47 and 29.37 ± 8.74 mL/kg/min, respectively. No difference in global mean GM perfusion was observed before (46.4 ± 11.4 mL/100 g/min) and after (50.0 ± 18.5 mL/100 g/min) exercise training. Voxel-by-voxel analysis identified four regions of higher CBF post-CR at cluster-level threshold of 10 contiguous voxels per cluster (*p* < 0.001, uncorrected). The regions, number of voxels and corresponding Talairach coordinates (X; Y; Z) were left AC (-1; 11; 26, 64 voxels), right AC (3; 27; 1, 25 voxels), right insula (45; 7; 4, 21 voxels) and left medial frontal gyrus (0; 46; -1, 52 voxels). After small volume correction, significant increase in CBF was found solely within the bilateral AC (*p* < 0.05, family wise error) as outlined in **Table 3** and **Figure 5**. Within the right AC recovery of CBF (∼30%) was observed post-CR in one area where a comparable decline in CBF compared to controls (∼33%) was observed at baseline, and a region where no change in GMV was observed after CR (**Figures 5** and **6**). There were no concomitant regional change in CBF and GMV after CR. No significant decrease in CBF over time was observed. Within regions of decreased CBF at baseline, a significant CBF increase was identified only within the right AC (**Figure 6**). However, higher GMV was found after CR in the bilateral pre- and post-central gyri and in the right superior frontal gyrus, while a sustained decrease in GMV over time was seen in the left superior temporal and right insular regions (**Figure 6**).

**Table 3:** Brain regions of increased CBF in patients post-CR (*N* = 17) from small volume correction analysis.

Brain region (Brodmann area)	Talairach coordinate (X, Y, Z)			Number of voxels	SPM t	FWE (*p*)
Left anterior cingulate (24)	-1	11	26	30	5.45	0.01
Right anterior cingulate (24)	5	27	1	16	4.90	0.02


*Brain areas and corresponding coordinates in anatomical Talairach space are listed. Results were corrected for multiple comparisons using small volume correction approach (p<0.05, FWE = family wise error).*

## Discussion

This study investigated the impact of cardiovascular disease in advancing age-related decline in cerebrovascular function and the mediatory role of aerobic exercise training. In a recent study, we described marked brain atrophy spanning the frontal, temporal and parietal lobes of CAD patients, and subsequent increase in brain volume with exercise (Anazodo et al., 2013). Here, in the same cohort of CAD patients, accelerated cerebrovascular decline was observed in several cognitive regions of the brain, independent of brain atrophy and recovery of perfusion in the right AC was demonstrated after 6 months of exercise training. Interestingly, these changes were greater than the magnitude of corresponding declines in brain volume or increase in brain volume after exercise, suggesting that the cerebrovascular function of certain regions of the brain in mid-life is highly sensitive to cardiovascular disease effects.

Normal aging is associated with increased plaque burden, arterial stiffness and endothelial dysfunction, all of which are in turn linked to reduced blood flow (Priebe, 2000). In the brain, by the age of 60, normal aging can reduce regional CBF by up to 15% (Bentourkia et al., 2000), offsetting the normal coupling of CBF to glucose metabolism (Bentourkia et al., 2000; Anazodo et al., 2015). This age-related compromise in CBF is exacerbated by the presence of cardiovascular disease, as demonstrated by our findings and supported by previous observations (de la Torre, 2000). Compared to age-matched controls, we found decreased CBF in the brains of CAD patients in regions considered stable in mid-life (Bentourkia et al., 2000; Chen et al., 2011) and known to be affected by various forms of vascular disease (for review see de la Torre, 2000 and Friedman et al., 2014). For instance, hypertension is associated with accelerated decline in regional perfusion in the orbitofrontal, AC and insular cortices (Beason-Held et al., 2007). In older adults with various forms of cardiovascular disease, reductions in frontal and temporal lobe CBF are associated with reduced regional cortical thickness and impairments in memory, attention and executive functions (Alosco et al., 2013). The decline in cognitive abilities in domains of executive function, attention, language, memory, and visuospatial skills seen in cardiovascular disease patients, independent of normal aging effects (Okonkwo et al., 2010), is consistent with the regional pattern of decreased resting CBF and CVR observed in these CAD patients. The accelerated decline in these cognitive domains and in global cognitive function could be related to vascular remodeling from atherosclerosis and arterial stiffening. Increased carotid intima media thickness, a marker of generalized atherosclerosis, is not only associated with increased risk for CAD (Iwamoto et al., 2012), but it has been linked to regional changes in CBF (Sojkova et al., 2010), increased risk for Alzheimer’s disease (Wendell et al., 2012) and accelerated decline in performance on tests for memory, language (semantic association fluency) and executive function, over time (Muller et al., 2007; Wendell et al., 2009). Interestingly, we found significantly higher carotid intima media thickness, decreased carotid artery compliance, an indication of arterial stiffening, as well as lower MoCA scores in CAD patients compared to controls. Arterial stiffness, even at the level of the aorta (Gauthier et al., 2015), and other vascular remodeling responses to vascular disease impair CVR (Lavi et al., 2006), which in turn can affect higher-order cognitive processes.

Cerebrovascular reactivity was significantly reduced in CAD patients in multiple regions including cognitive control centers in the AC, superior frontal and post-central gyrus. Typically, CBF reductions are likely either a result of reduced supply to tissue, signifying vascular dysfunction, or a result of reduced metabolic demand, signifying a decline in neuronal activity. The observed reduction in CVR in areas of reduced CBF in the CAD patients supports the former since it indicates that the brain’s vascular response to a vasoactive stimulus was impaired, suggesting that the CBF response to increased functional activation could also be impaired (Stefanovic et al., 2008). A recent study in older adults with type 2 diabetes mellitus found that decreased regional CVR was associated with greater decline in executive function, and the higher the levels of serum inflammatory markers, the greater the CVR impairment (Chung et al., 2015). It has been suggested that impaired CVR could reveal cognitive dysfunction sooner and with greater sensitivity than resting CBF. Lu et al. (2011) demonstrated that in the prefrontal cortex, CVR showed an accelerated decline with age compared to CBF. These observations underscore the significance of CVR as a marker of cerebrovascular health and potentially of cognitive function in cardiovascular disease patients.

One of the main findings of the current study was the observation of differences in the spatial patterns of hypoperfusion and brain atrophy in the CAD patients. Of the 11 clusters identified as having lower basal CBF, only four clusters- all within the prefrontal cortex- showed a concurrent decrease in GMV following BPM analysis. A spatial mismatch between regional hypoperfusion and atrophy has also been observed in normal aging (Chen et al., 2011). These observations suggest that certain areas of the brain respond differently to hemodynamic and structural changes associated with aging and vascular disease. It is also possible that the CBF changes observed in CAD patients could precede structural changes. It is important to consider that the poor spatial resolution of ASL compared to structural imaging could lead to partial volume errors. To minimize this, differences in CBF between patients and controls were only conducted in voxels that were classified as being comprised of at least 80% GM, followed by BPM analysis to control for GMV changes. An alternative approach would be to use linear regression methods such as outlined by Asllani et al. (2008) to extract a GM CBF image. However, this approach can introduce substantial spatial blurring, which when applied to regions as small as those identified in the current study, could eliminate the observed effects.

Other notable findings of the current study were increased CBF in the bilateral AC and recovery of CBF in the right AC after mild-to-moderate aerobic fitness training. This finding is consistent with evidence of robust improvements in structure and function of the AC in older adults without CAD after aerobic exercise training. Higher CBF in the AC cortex has been observed in aerobically trained older adults compared to sedentary controls (Chapman et al., 2013). Increased GMV has also been observed in the dorsal AC of older adults that participated in 6 months of aerobic training compared to a control group that only performed toning and stretching exercises (Colcombe et al., 2006). Burdette et al. (2010) found greater functional connectivity between the hippocampus and AC cortex of older healthy adults in an exercise training group. Further, in older adults, it appears that having a higher cardiorespiratory fitness level is associated with greater WM integrity in the middle cingulate cortex (Marks et al., 2011) and greater activation of the AC during dual-task processing (Wong et al., 2015). A recent study also reported a positive correlation between CBF in the AC and VO_2_ max in CAD patients (MacIntosh et al., 2014). Conversely, Colcombe et al. (2004) associated the level of aerobic fitness with decreased activity in the AC and increased activity in the middle frontal, superior frontal, and parietal regions in older adults performing conflicting response tasks. Though they attributed their observation to improved adaptations in the attentional network following exercise training (Colcombe et al., 2004), it could also signify the effects of exercise in enhancing both cognitive and cardiovascular control (Shoemaker et al., 2012). Decreased activity in the AC (subgenual) and medial prefrontal cortex, along with increased activity in the insular cortex, are strongly associated with cardiovascular adjustments to acute stressors during physical, cognitive and emotional demands (Shoemaker et al., 2012). Although we did not find significant changes in CBF in the insular or prefrontal regions after exercise training, we previously reported increased brain volume in the medial prefrontal region in addition to other cognitive areas (Anazodo et al., 2013), which are consistently associated with improved higher cardiorespiratory fitness level (Hayes et al., 2013). The recovery of CBF in the right and not the left AC could follow the normal morphological asymmetry and functional specialization of the AC, where the right AC has greater GMV and more structural and functional connectivity than the left AC (Yan et al., 2009). Similar to Chapman et al. (2013), we observed no increase in global CBF after exercise training; however, the effects of exercise on global CBF are conflicting (Ogoh and Ainslie, 2009). We were unable to investigate regional CVR response to exercise training given the relatively small sample, a global increase in CVR has been reported in older adults following 12 weeks of mild-to-moderate aerobic exercise (Murrell et al., 2013) and in stroke patients (Ivey et al., 2011) after completing a 6-months exercise program. In general, our findings of recovery of regional CBF in the AC cortex of CAD patients following CR is noteworthy given the central role of the AC in executive function (Allman et al., 2001) and the strong association between lower executive function and increased risk of cardiovascular disease (Okonkwo et al., 2010; Rostamian et al., 2015). This finding supports the role of aerobic fitness in preservation of brain health following injury.

### Study Considerations

The findings presented in the current study although largely attributed to CAD, could also be associated with lifestyle factors (Friedman et al., 2014). Compared to controls, CAD patients had significantly lower fitness levels and were generally overweight. The effect of these lifestyle factors on cerebral perfusion and brain structure in the presence of vascular disease are yet to be determined. A large majority of the CAD patients were on antihypertensive and hypolipidemic drug. These agents are known to affect cerebrovascular hemodynamics and could mask vascular disease effects or attenuate exercise effects. Statins for instance are hypolipidemic agents associated with increased CBF and vascular reactivity (Giannopoulos et al., 2014), while antihypertensive drugs such as beta-blockers can limit submaximal exercise capacity (van Baak et al., 1987). At our institution, aggressive early revascularization is the standard mode of care for CAD. This coupled with the relatively long period of up to 3 months between patient referral, evaluation at the cardiac center, intake at the CR program, and start of this study, could explain the lack of significant differences in baseline cardiovascular function between patients and controls. Furthermore, we did not explore potential ongoing cerebrovascular disease in the study population, specifically, WM lesions, which could further contribute to cerebral hypoperfusion and atrophy. Our analysis, however, focused on vascular disease effects within GM. Areas associated with WM hyperintensities are typically deep within WM regions.

Other study considerations include the inherent limitations with the ASL imaging and the hypercapnic challenge. First, the 2D EPI readout used in this study for ASL imaging, limited data acquisition to slices superior to the anterior commissure – posterior commissure line. As such, regions near tissue-air/bone interfaces prone to increased susceptibility artifacts were excluded. Cerebral hypoperfusion may well exist in the inferior regions of the frontal and parietal lobes, since brain atrophy has been observed in these regions in the same cohort of CAD patients (Anazodo et al., 2013). Second, our choice of post-labeling delay was relatively short. It was selected to mediate the effects of short arterial transit time at high flow velocities during hypercapnia and increase SNR. This resulted in a tSNR comparable to newer ASL methods using three-dimensional gradient-spin echo (Günther et al., 2005) readouts, where whole brain coverage can be achieved at longer post-label delay and minimal susceptibility issues. A short post-labeling delay could result in an underestimation of CBF in patients with longer transit times, however, no watershed artifacts, which are typical of transit time errors, were observed in the CBF images (**Figure 2**). Lastly, the CVR results were limited by a small sample size due to poor compliance with the hypercapnic manipulations, compounded by inter-subject variability in ventilation response during CO_2_ inhalation. Reproducible changes in P_ET_CO_2_ were achieved by controlling the respiration rate using a metronome. However, commercial devices designed to control P_ET_CO_2_ breath by breath, such as the RespirAct (Fierstra et al., 2013) would improve the sensitivity of CVR measurements.

## Conclusion

This study represents a concerted attempt within a single cohort to investigate the association between cardiovascular disease and cerebral hemodynamics, as well as investigating the potential benefits of physical activity. The findings demonstrated a region-specific vulnerability to cardiovascular disease that appeared to accelerate the normal changes in CBF associated with aging. It is possible that reductions in regional CBF and CVR, related to vascular disease could drive cortical decline. However, the fundamental mechanisms underlining the association between vascular disease and cortical decline remain unclear and require further investigation. The ability of an aerobic fitness program designed for CAD patients to improve cerebral perfusion was demonstrated in a relatively small sample. This work sets the stage for future investigations in larger cohorts of vascular disease patients to understand how cardiorespiratory fitness training impacts cerebrovascular hemodynamics.

## Author Contributions

Conceived and designed the experiments: JS, KSt.L, NS, and UA. Performed the experiments: UA. Analyzed the data: UA, KSt.L, and JS. Interpreted data: UA, KSt.L, JS, and NS. Contributed reagents/materials/analysis tools; DW and TS. Wrote the paper: UA, KSt.L, and JS.

## Conflict of Interest Statement

The authors declare that the research was conducted in the absence of any commercial or financial relationships that could be construed as a potential conflict of interest.

## Footnotes

1 http://www.fil.ion.ucl.ac.uk

2 http://dbm.neuro.uni-jena.de/vbm

3 http://dbm.neuro.uni-jena.de/vbm/

4 http://marsbar.sourceforge.net

## References

[B1] Allman J. M. Hakeem A. Erwin J. M. Nimchinsky E. Hof P. (2001). The anterior cingulate cortex. *Ann. N. Y. Acad. Sci.* 935 107–117. 10.1111/j.1749-6632.2001.tb03476.x 11411161

[B2] Alosco M. L. Gunstad J. Jerskey B. A. Xu X. Clark U. S. Hassenstab J. (2013). The adverse effects of reduced cerebral perfusion on cognition and brain structure in older adults with cardiovascular disease. *Brain Behav.* 3 626–636. 10.1002/brb3.171 24363966 PMC3868168

[B3] Anazodo U. C. Shoemaker J. K. Suskin N. St. Lawrence K. S. (2013). An investigation of changes in regional gray matter volume in cardiovascular disease patients, pre and post cardiovascular rehabilitation. *Neuroimage Clin.* 3 388–395. 10.1016/j.nicl.2013.09.011 24273722 PMC3814972

[B4] Anazodo U. C. Thiessen J. D. Ssali T. Mandel J. Günther M. Butler J. (2015). Feasibility of simultaneous whole-brain imaging on an integrated PET-MRI system using an enhanced 2-point Dixon attenuation correction method. *Front. Neurosci.* 8:434 10.3389/fnins.2014.00434 PMC4283546 25601825

[B5] Asllani I. Borogovac A. Brown T. R. (2008). Regression algorithm correcting for partial volume effects in arterial spin labeling MRI. *Magn. Reson. Med.* 60 1362–1371. 10.1002/mrm.21670 18828149

[B6] Beason-Held L. L. Moghekar A. Zonderman A. B. Kraut M. A. Resnick S. M. (2007). Longitudinal changes in cerebral blood flow in the older hypertensive brain. *Stroke* 38 1766–1773. 10.1161/STROKEAHA.106.477109 17510458

[B7] Bentourkia M. Bol A. Ivanoiu A. Labar D. Sibomana M. Coppens A. (2000). Comparison of regional cerebral blood flow and glucose metabolism in the normal brain: effect of aging. *J. Neurol. Sci.* 181 19–28. 10.1016/S0022-510X(00)00396-8 11099707

[B8] Burdette J. H. Laurienti P. J. Espeland M. A. Morgan A. Telesford Q. Vechlekar C. D. (2010). Using network science to evaluate exercise-associated brain changes in older adults. *Front. Aging Neurosci.* 2:23 10.3389/fnagi.2010.00023 PMC2893375 20589103

[B9] Calhoun D. A. Jones D. Textor S. Goff D. C. Murphy T. P. Toto R. D. (2008). Resistant hypertension: diagnosis, evaluation, and treatment: a scientific statement from the american heart association professional education committee of the council for high blood pressure research. *Circulation* 117 e510–e526. 10.1161/CIRCULATIONAHA.108.189141 18574054

[B10] Casanova R. Srikanth R. Baer A. Laurienti P. J. Burdette J. H. Hayasaka S. (2007). Biological parametric mapping: a statistical toolbox for multimodality brain image analysis. *Neuroimage* 34 137–143. 10.1016/j.neuroimage.2006.09.011 17070709 PMC1994117

[B11] Chapman S. B. Aslan S. Spence J. S. Defina L. F. Keebler M. W. Didehbani N. (2013). Shorter term aerobic exercise improves brain, cognition, and cardiovascular fitness in aging. *Front. Aging Neurosci.* 5:75 10.3389/fnagi.2013.00075 PMC3825180 24282403

[B12] Chen J. J. Rosas H. D. Salat D. H. (2011). Age-associated reductions in cerebral blood flow are independent from regional atrophy. *Neuroimage* 55 468–478. 10.1016/j.neuroimage.2010.12.032 21167947 PMC3435846

[B13] Chung C.-C. Pimentel D. Jor’dan A. J. Hao Y. Milberg W. Novak V. (2015). Inflammation-associated declines in cerebral vasoreactivity and cognition in type 2 diabetes. *Neurology* 85 450–458. 10.1212/WNL.0000000000001820 26156513 PMC4534073

[B14] Clark A. M. Hartling L. Vandermeer B. McAlister F. A. (2005). Meta-analysis: secondary prevention programs for patients with coronary artery disease. *Ann. Intern. Med.* 143 659–672. 10.7326/0003-4819-143-9-200511010-00010 16263889

[B15] Colcombe S. J. Erickson K. I. Scalf P. E. Kim J. S. Prakash R. Mcauley E. (2006). Aerobic exercise training increases brain volume in aging humans. *J. Geronotol.* 61 1166–1170. 10.1093/gerona/61.11.1166 17167157

[B16] Colcombe S. J. Kramer A. F. Erickson K. I. Scalf P. McAuley E. Cohen N. J. (2004). Cardiovascular fitness, cortical plasticity, and aging. *Proc. Natl. Acad. Sci. U.S.A.* 101 3316–3321. 10.1073/pnas.0400266101 14978288 PMC373255

[B17] de la Torre J. (2000). Critically attained threshold of cerebral hypoperfusion: the CATCH hypothesis of Alzheimer’s pathogenesis. *Neurobiol. Aging* 21 331–342. 10.1016/S0197-4580(00)00111-1 10867218

[B18] de Toledo Ferraz Alves T. C. Ferreira L. K. Busatto G. F. (2010). Vascular diseases and old age mental disorders: an update of neuroimaging findings. *Curr. Opin. Psychiatry* 23 491–497. 10.1097/YCO.0b013e32833e339c 20733494

[B19] Fierstra J. Sobczyk O. Battisti-Charbonney A. Mandell D. M. Poublanc J. Crawley A. P. (2013). Measuring cerebrovascular reactivity: what stimulus to use? *J. Physiol.* 591 5809–5821. 10.1113/jphysiol.2013.259150 24081155 PMC3872753

[B20] Friedman J. I. Tang C. Y. de Haas H. J. Changchien L. Goliasch G. Dabas P. (2014). Brain imaging changes associated with risk factors for cardiovascular and cerebrovascular disease in asymptomatic patients. *JACC Cardiovasc. Imaging* 7 1039–1053. 10.1016/j.jcmg.2014.06.014 25323165

[B21] Gauthier C. J. Lefort M. Mekary S. Desjardins-Crépeau L. Skimminge A. Iversen P. (2015). Hearts and minds: linking vascular rigidity and aerobic fitness with cognitive aging. *Neurobiol. Aging* 36 304–314. 10.1016/j.neurobiolaging.2014.08.018 25308963

[B22] Giannopoulos S. Katsanos A. H. Kosmidou M. Tsivgoulis G. (2014). Statins and vascular dementia: a review. *J. Alzheimers. Dis.* 42(Suppl. 3), S315–S320. 10.3233/JAD-132366 24662103

[B23] Günther M. Oshio K. Feinberg D. A. (2005). Single-shot 3D imaging techniques improve arterial spin labeling perfusion measurements. *Magn. Reson. Med.* 54 491–498. 10.1002/mrm.20580 16032686

[B24] Hayes S. M. Alosco M. L. Forman D. E. (2014). The effects of aerobic exercise on cognitive and neural decline in aging and cardiovascular disease. *Curr. Geriatr. Rep.* 3 282–290. 10.1007/s13670-014-0101-x 25750853 PMC4349343

[B25] Hayes S. M. Hayes J. P. Cadden M. Verfaellie M. (2013). A review of cardiorespiratory fitness-related neuroplasticity in the aging brain. *Front. Aging Neurosci.* 5:31 10.3389/fnagi.2013.00031 PMC3709413 23874299

[B26] Howlader N. Noone A. M. Krapcho M. Grashell J. Neyman N. Alterkruse S. F. (eds) (2013). *SEER Cancer Statistics Review, 1975-2010*. Bethesda, MD: National Cancer Institute.

[B27] Ivey F. M. Ryan A. S. Hafer-Macko C. E. Macko R. F. (2011). Improved cerebral vasomotor reactivity after exercise training in hemiparetic stroke survivors. *Stroke* 42 1994–2000. 10.1161/STROKEAHA.110.607879 21636819

[B28] Iwamoto Y. Maruhashi T. Fujii Y. Idei N. Fujimura N. Mikami S. (2012). Intima-media thickness of brachial artery, vascular function, and cardiovascular risk factors. *Arterioscler. Thromb. Vasc. Biol.* 32 2295–2303. 10.1161/ATVBAHA.112.249680 22796580

[B29] Lavi S. Gaitini D. Milloul V. Jacob G. (2006). Impaired cerebral CO_2_ vasoreactivity: association with endothelial dysfunction. *Am. J. Physiol. Heart Circ. Physiol.* 291 H1856–H1861. 10.1152/ajpheart.00014.2006 16766649

[B30] Lloyd-Jones D. M. Larson M. G. Beiser A. Levy D. (1999). Lifetime risk of developing coronary heart disease. *Lancet* 353 89–92. 10.1016/S0140-6736(98)10279-9 10023892

[B31] Lloyd-Jones D. M. Leip E. P. Larson M. G. D’Agostino R. B. Beiser A. Wilson P. W. F. (2006). Prediction of lifetime risk for cardiovascular disease by risk factor burden at 50 years of age. *Circulation* 113 791–798. 10.1161/CIRCULATIONAHA.105.548206 16461820

[B32] Lu H. Xu F. Rodrigue K. M. Kennedy K. M. Cheng Y. Flicker B. (2011). Alterations in cerebral metabolic rate and blood supply across the adult lifespan. *Cereb. Cortex* 21 1426–1434. 10.1093/cercor/bhq224 21051551 PMC3097991

[B33] MacIntosh B. J. Swardfager W. Crane D. E. Ranepura N. Saleem M. Oh P. I. (2014). Cardiopulmonary fitness correlates with regional cerebral grey matter perfusion and density in men with coronary artery disease. *PLoS ONE* 9:e91251 10.1371/journal.pone.0091251 PMC3951327 24622163

[B34] Maldjian J. A. Laurienti P. J. Kraft R. A. Burdette J. H. (2003). An automated method for neuroanatomic and cytoarchitectonic atlas-based interrogation of fMRI data sets. *Neuroimage* 19 1233–1239. 10.1016/S1053-8119(03)00169-1 12880848

[B35] Mampuya W. M. (2012). Cardiac rehabilitation past, present and future: an overview. *Cardiovasc. Diagn. Ther.* 2 38–49. 10.3978/j.issn.2223-3652.2012.01.02 24282695 PMC3839175

[B36] Marks B. L. Katz L. M. Styner M. Smith J. K. (2011). Aerobic fitness and obesity: relationship to cerebral white matter integrity in the brain of active and sedentary older adults. *Br. J. Sport. Med.* 45 1208–1215. 10.1136/bjsm.2009.068114 20558529

[B37] Muller M. Grobbee D. E. Aleman A. Bots M. van der Schouw Y. T. (2007). Cardiovascular disease and cognitive performance in middle-aged and elderly men. *Atherosclerosis* 190 143–149. 10.1016/j.atherosclerosis.2006.01.005 16488420

[B38] Murrell C. J. Cotter J. D. Thomas K. N. Lucas S. J. E. Williams M. J. A. Ainslie P. N. (2013). Cerebral blood flow and cerebrovascular reactivity at rest and during sub-maximal exercise: effect of age and 12-week exercise training. *Age (Omaha)* 35 905–920. 10.1007/s11357-012-9414-x PMC3636405 22669592

[B39] Nasreddine Z. Phillips N. Bedirian V. Charbonneau S. Whitehead V. Collin I. (2005). The montreal cognitive assessment, MoCA: a brief screening tool for mild cognitive impairment. *J. Am. Geriatr. Soc.* 53 695–699. 10.1111/j.1532-5415.2005.53221.x 15817019

[B40] Nielson C. A. Frances M. F. Fitzgeorge L. Prapavessis H. Zamir M. Shoemaker J. K. (2014). Impact of a smoking cessation lifestyle intervention on vascular mechanics in young women. *Appl. Physiol. Nutr. Metab.* 580 572–580. 10.1139/apnm-2013-0272 24766240

[B41] Novack B. P. Shenkin H. A. Bortin L. Goluboff B. Soffe A. M. (1953). Effects of carbon dioxide inhalation upon the cerebral blood flow and cerbral oxygen consumption in vascular disease. *J. Clin. Invest.* 32 696–702. 10.1172/JCI102783 13069617 PMC438392

[B42] Ogoh S. Ainslie P. N. (2009). Cerebral blood flow during exercise: mechanisms of regulation. *J. Appl. Physiol.* 107 1370–1380. 10.1152/japplphysiol.00573.2009 19729591

[B43] Okonkwo O. C. Cohen R. A. Gunstad J. Tremont G. Alosco M. L. Poppas A. (2010). Longitudinal trajectories of cognitive decline among older adults with cardiovascular disease. *Cerebrovasc. Dis.* 30 362–373. 10.1159/000319564 20693791 PMC3014862

[B44] Priebe H.-J. (2000). The aged cardiovascular risk patient. *Br. J. Anaesth.* 85 763–778. 10.1093/bja/85.5.763 11094595

[B45] Rasmussen L. S. (2006). Postoperative cognitive dysfunction: incidence and prevention. *Best Pract. Res. Clin. Anaesthesiol.* 20 315–330. 10.1016/j.bpa.2005.10.011 16850780

[B46] Rostamian S. van Buchem M. A. Westendorp R. G. J. Jukema J. W. Mooijaart S. P. Sabayan B. (2015). Executive function, but not memory, associates with incident coronary heart disease and stroke. *Neurology* 85 783–789. 10.1212/WNL.0000000000001895 26245926

[B47] Shoemaker J. K. Wong S. W. Cechetto D. F. (2012). Cortical circuitry associated with reflex cardiovascular control in humans: does the cortical autonomic network “speak” or “listen” during cardiovascular arousal. *Anat. Rec.* 295 1375–1384. 10.1002/ar.22528 22848047

[B48] Sojkova J. Najjar S. S. Beason-Held L. L. Metter E. J. Davatzikos C. Kraut M. A. (2010). Intima-media thickness and regional cerebral blood flow in older adults. *Stroke* 41 273–279. 10.1161/STROKEAHA.109.566810 20044526 PMC2853882

[B49] Stefanovic B. Hutchinson E. Yakovleva V. Schram V. Russell J. T. Belluscio L. (2008). Functional reactivity of cerebral capillaries. *J. Cereb. Blood Flow Metab.* 28 961–972. 10.1038/sj.jcbfm.9600590 18059431 PMC3197804

[B50] Tancredi F. B. Lajoie I. Hoge R. D. (2015). Test-retest reliability of cerebral blood flow and blood oxygenation level-dependent responses to hypercapnia and hyperoxia using dual-echo pseudo-continuous arterial spin labeling and step changes in the fractional composition of inspired gases. *J. Magn. Reson. Imaging* 42 1144–1157. 10.1002/jmri.24878 25752936

[B51] Tzourio-Mazoyer N. Landeau B. Papathanassiou D. Crivello F. Etard O. Delcroix N. (2002). Automated anatomical labeling of activations in SPM using a macroscopic anatomical parcellation of the MNI MRI single-subject brain. *Neuroimage* 15 273–289. 10.1006/nimg.2001.0978 11771995

[B52] van Baak M. A. Böhm R. O. Arends B. G. van Hooff M. E. Rahn K. H. (1987). Long-term antihypertensive therapy with beta-blockers: submaximal exercise capacity and metabolic effects during exercise. *Int. J. Sports Med.* 8 342–347. 10.1055/s-2008-1025681 2890591

[B53] Wang J. Alsop D. C. Song H. K. Maldjian J. A. Tang K. Salvucci A. E. (2003). Arterial transit time imaging with flow encoding arterial spin tagging (FEAST). *Magn. Reson. Med.* 50 599–607. 10.1002/mrm.10559 12939768

[B54] Wendell C. R. Waldstein S. R. Ferrucci L. O’Brien R. J. Strait J. B. Zonderman A. B. (2012). Carotid atherosclerosis and prospective risk of dementia. *Stroke* 43 3319–3324. 10.1161/STROKEAHA.112.672527 23103489 PMC3508298

[B55] Wendell C. R. Zonderman A. B. Metter E. J. Najjar S. S. Waldstein S. R. (2009). Carotid intimal medial thickness predicts cognitive decline among adults without clinical vascular disease. *Stroke* 40 3180–3185. 10.1161/STROKEAHA.109.557280 19644063 PMC2753681

[B56] Wong C. N. Chaddock-Heyman L. Voss M. W. Burzynska A. Z. Basak C. Erickson K. I. (2015). Brain activation during dual-task processing is associated with cardiorespiratory fitness and performance in older adults. *Front. Aging Neurosci.* 7:154 10.3389/fnagi.2015.00154 PMC4532928 26321949

[B57] Xu G. Rowley H. A. H. Wu G. Alsop D. C. Shankaranarayanan A. Dowling M. (2010). Reliability and precision of pseudo-continuous arterial spin labeling perfusion MRI on 3.0 T and comparison with 15O-water PET in elderly subjects at risk for Alzheimer’s disease. *NMR Biomed.* 23 286–293. 10.1002/nbm.1462.Reliability 19953503 PMC2843795

[B58] Yan H. Zuo X.-N. Wang D. Wang J. Zhu C. Milham M. P. (2009). Hemispheric asymmetry in cognitive division of anterior cingulate cortex: a resting-state functional connectivity study. *Neuroimage* 47 1579–1589. 10.1016/j.neuroimage.2009.05.080 19501172

